# 20例多中心型Castleman病患者的临床特征及预后分析

**DOI:** 10.3760/cma.j.issn.0253-2727.2021.06.012

**Published:** 2021-06

**Authors:** 慧守 樊, 文强 严, 婧钰 许, 佳慧 刘, 辰星 杜, 伟薇 隋, 书会 邓, 薇 刘, 树华 易, 文阳 黄, 燕 徐, 耀中 赵, 录贵 邱, 德慧 邹, 刚 安

**Affiliations:** 中国医学科学院血液病医院（中国医学科学院血液学研究所），实验血液学国家重点实验室，国家血液系统疾病临床医学研究中心 State Key Laboratory of Experimental Hematology, National Clinical Research Center for Blood Diseases, Institute of Hematology & Blood Diseases Hospital, Chinese Academy of Medical Sciences & Peking Union Medical College, Tianjin 300020, China

Castleman病（Castleman disease，CD）是一类临床表现高度异质性、具有特征性病理改变的淋巴组织增殖性疾病。临床上根据淋巴结分布和器官受累部位的情况将CD进一步分为单中心型Castleman病（UCD）和多中心型Castleman病（MCD）。UCD患者手术切除病灶后多预后良好，而MCD患者多需要全身治疗，我们在此回顾性分析2013年8月至2020年8月在我中心诊断的20例MCD患者的临床特征、治疗方案及预后。

## 病例与方法

1. 病例：入选病例为2013年8月至2020年8月我院淋巴瘤诊疗中心诊断的20例MCD患者。所有患者均有完整的临床和病理资料。收集患者初诊时年龄、性别、B症状、受累淋巴结情况、细胞因子水平、LDH水平、β_2_微球蛋白水平、颈胸腹盆CT、淋巴结活检、骨髓涂片及病理、治疗方案等基础资料。

2. 诊断标准：MCD诊断标准为多部位淋巴结肿大，具有特征性的组织病理学改变，有多系统器官受累表现，排除肿瘤、风湿免疫及感染等其他原发病[1]。

3. 疾病分型：所有患者临床分型均为MCD。因HHV-8、组织病理标本的EB病毒编码RNA（Epstein-Barr encoded-RNA，EBER）染色及潜伏期相关核抗原（latency-asso-ciated nuclear antigen，LANA-1）染色非常规检查，故未再对MCD患者进一步分型。

根据组织病理学特点[2]，CD可分为：①透明血管型（HV）；②浆细胞型（PC）；③混合型（MIX）。

4. 治疗：2例患者自明确诊断后拒绝治疗失访。4例患者自明确诊断至今未启动治疗（随访时诉平素监测血常规示HGB波动于70～90 g/L，不影响正常生活）。在启动治疗的14例患者中，传统化疗组（含或不含CD20单抗）患者6例，其中采用CHOP（环磷酰胺+多柔比星+长春地辛+泼尼松）方案2例，R-CHOP（CD20单抗+CHOP）方案1例，VDDC（硼替佐米+脂质体阿霉素+地塞米松+环磷酰胺）方案1例，GDP（吉西他滨+地塞米松+顺铂）方案1例，苯丁酸氮芥片联合泼尼松方案1例。免疫调节治疗组患者6例：2例接受TP（沙利度胺+地塞米松）方案（1例患者初始即应用TP方案，1例患者因口服TCP方案1周后出现皮疹反应，改为TP方案），4例患者接受TCP方案（沙利度胺100 mg，1次/晚；环磷酰胺300 mg/m^2^，每周1次；泼尼松1 mg/kg，每周2次），应用TCP方案的患者均同时口服阿司匹林预防血栓形成。接受自体造血干细胞移植治疗2例，其中R-GDPE（CD20单抗+吉西他滨+地塞米松+顺铂+依托泊苷）方案4个疗程后移植1例，环磷酰胺+G-CSF方案动态动员后采集外周血造血干细胞后移植1例。

5. 疗效评估：疗效评价包括完全缓解（CR），部分缓解（PR），疾病稳定（SD），复发或进展（PD）[1]。

6. 随访：所有入选患者均接受随访。随访截止时间为2020年11月19日，随访方式为电话随访。无进展生存（PFS）时间定义为疾病诊断至疾病进展或末次随访的间隔时间。总生存（OS）时间定义为自疾病诊断至临床死亡或末次随访的间隔时间。中位随访时间为36（19～54）个月。

7. 统计学处理：采用SPSS 25.0软件进行统计学分析，连续变量采用中位数（范围）表示。采用Kaplan-Meier曲线进行预后分析，以Log-rank检验进行组间比较，*P*<0.05为差异有统计学意义。

## 结果

一、临床特征

20例MCD患者的临床特征见表1。除主要临床表现外，其他临床表现还包括血小板增多（20％）、肝脾肿大（15％）、肢端麻木（10％）、球蛋白升高（5％）、泡沫尿（5％）、腰背疼痛（5％）、黄疸（5％）等。肿大淋巴结常见的累及部位依次为颈部（90％）、腹股沟（55％）、腋下（50％）、腹膜后（50％）、腹腔（40％）、盆腔（35％）、纵隔6例（30％）等。

**表1:** 20例多中心型Castleman病患者的临床特征

临床特征	数值
性别［例（％）］	
男	14（70）
女	6（30）
中位年龄［岁，*M*（范围）］	46（15～70）
主要临床表现［例（％）］	
淋巴结肿大或发现肿物	12（60）
B症状	12（60）
贫血	14（70）
低白蛋白血症	13（65）
多克隆免疫球蛋白升高	11（55）
其他系统受累［例（％）］	
肾功能不全^a^	12（92）
肺部间质性病变	7（35）
肢体水肿/浆膜腔积液	3（15）
病理分型［例（％）］	
浆细胞型	16（80）
透明血管型	2（10）
混合型	2（10）
治疗方式^b^［例（％）］	
传统化疗	6（43）
免疫调节治疗	6（43）
自体造血干细胞移植	2（14）

注：B症状：发热、盗汗、体重减轻；^a^肾小球滤过率<60 ml·min^−1^·1.73m^−2^或蛋白尿（总蛋白>150 mg/24 h），^a^13例患者有肾功能资料；^b^14例患者启动治疗

二、实验室检查

1. 常规实验室检查：20例MCD患者的中位WBC 5.96（3.43～17.80）×10^9^/L，中位HGB 110（71.0～168.0）g/L，中位PLT 284（115～696）×10^9^/L，中位ANC 3.73（1.65～9.38）×10^9^/L，中位淋巴细胞1.45（0.81～5.87）×10^9^/L，中位单核细胞0.45（0.16～1.43）×10^9^/L。中位总蛋白（TP）83.95（59.5～138.0）g/L。多克隆免疫球蛋白升高发生率55％（11/20），白蛋白（ALB）减少发生率65％（13/20），24 h尿蛋白升高发生率92％（11/12），中位肾小球滤过率（eGFR）为95.5（43.7～229.0）ml·min^−1^·1.73m^−2^，血β_2_-微球蛋白增高发生率88％（15/17），LDH升高发生率5％（1/20），C反应蛋白（CRP）增高发生率91％（10/11），中位CRP水平为85.8（1～212）mg/L，红细胞生成素（EPO）升高发生率89％（8/9），乙肝表面抗原阳性发生率5％（1/20）。患者血、尿免疫固定电泳均为阴性且均无明确的风湿免疫、严重感染、恶性肿瘤等相关疾病。

2. 病毒筛查：2例患者EBV抗体阳性，EBV拷贝数均低于正常检测范围上限（正常范围<1000.0拷贝/ml）；巨细胞病毒（CMV）抗体IgM、CMV抗体IgG、单纯疱疹病毒（HSV）-Ⅰ型抗体IgM、HSV-Ⅰ型抗体IgG、HSV-Ⅱ型抗体IgM、HSV-Ⅱ型抗体IgG阳性患者的比例分别为19％（3/16）、77％（10/13）、19％（3/16）、92％（12/13）、13％（2/16）、8％（1/13）。

3. 细胞因子水平：在接受细胞因子水平检测的MCD患者中，所有患者都至少有一种细胞因子水平升高，其中中位IL-6水平为69.8（2.0～147.9）pg/ml。≥3种细胞因子水平升高的比例为54％（7/13）。各类细胞因子升高发生率分别为IL-2R 80％（8/10），IL-6 77％（10/13），TNFα 64％（7/11），IL-8 42％（5/12），IL-10 33％（4/12），IL-1β 33％（3/9）。

4. 病理：MCD患者淋巴结病理最常见的免疫表型为CD3（+），CD5（+），CD10（+），CD21FDC（+），CD30（+），CD138（+），PAX5（+），Bcl-2（−/+），Bcl-6（+），Kappa（+），Lambda（+），CyclinD1（−），Ki-67（+），IgG（+），IgG4（+，<40％）。

17例患者行骨髓病理检查，其中骨髓增生明显活跃、活跃、减低、重度减低的患者分别占23％（4例）、53％（9例）、18％（3例）、6％（1例）。骨髓纤维染色为阴性、1级、2级的患者分别占65％（11例）、29％（5例）、6％（1例）。骨髓免疫组化常见的表型为CD3（+），CD5（+），PAX5（+），CD34（−），CD56（−/弱表达），CD138（+），Kappa（+），Lambda（+）。

三、治疗疗效及生存分析

MCD患者接受的治疗方案和治疗结果见表2。

**表2:** 20例多中心型Castleman病患者的治疗、疗效及随访结果

例号	性别	年龄（岁）	病理类型	治疗情况	最佳疗效	是否进展	无进展生存（月）	是否死亡	生存时间（月）
1	男	66	PC	口服苯丁酸氮芥片+泼尼松	NA	是	36	是	36
2	男	48	PC	TP方案口服	PR	否	23	否	23
3	男	50	PC	R-CHOP方案×6个疗程→TD方案×3个疗程→托珠单抗+环孢素+泼尼松×5个疗程	SD	是	22	否	45
4	男	62	PC	继续随访		否	75	否	75
5	男	62	PC	TCP方案	PR	否	3	否	3
6	男	33	PC	VDDC方案×7个疗程	PR	否	59	否	59
7	男	32	PC	TCP方案	PR	否	4	否	4
8	男	37	PC	CHOP×5个疗程停疗	CR	否	29	否	29
9	男	70	PC	继续随访		否	40	否	40
10	男	37	HV	CTX+G-CSF采干→ASCT	CR	否	37	否	37
11	男	67	PC	继续随访		否	81	否	81
12	女	28	MIX	失访	NA	NA	NA	NA	NA
13	女	29	MIX	R-GDPE方案×4个疗程→ASCT	CR	否	53	否	53
14	男	45	PC	TP方案	PR	是	38	否	47
15	女	35	HV	失访	NA	NA	NA	NA	NA
16	女	47	PC	继续随访		否	19	否	19
17	男	51	PC	CHOP方案×5个疗程	NA	是	12	是	12
18	女	48	PC	GDP方案×6个疗程	CR	否	87	否	87
19	男	39	PC	TCP方案	PR	否	15	否	15
20	女	15	PC	TCP方案	SD	否	7	否	7

注：PC：浆细胞型；HV：透明血管型；MIX：混合型；CHOP：环磷酰胺+多柔比星+长春地辛+泼尼松；R-CHOP：CD20单抗+环磷酰胺+多柔比星+长春地辛+泼尼松；VDDC：硼替佐米+脂质体阿霉素+地塞米松+环磷酰胺；GDP：吉西他滨+地塞米松+顺铂；R-GDPE：美罗华+吉西他滨+地塞米松+顺铂+依托泊苷；TD：沙利度胺+地塞米松；TP：沙利度胺+地塞米松；TCP：沙利度胺+环磷酰胺+地塞米松；CTX：环磷酰胺；G-CSF：粒细胞集落刺激因子；ASCT：自体造血干细胞移植；CR：完全缓解；PR：部分缓解；SD：疾病稳定；PD：疾病进展；NA：不可得知

在启动治疗的14例MCD患者中，因自体造血干细胞移植组病例数较少，故进一步分析传统化疗组和免疫调节治疗组的12例患者。传统化疗组和免疫调节治疗组患者的客观缓解率（ORR）分别为75％（3/4）、83％（5/6）。12例患者的中位PFS时间为37.8个月，两组患者的中位PFS时间分别为36.2和37.8个月，差异无统计学意义（*P*＝0.825）。12例患者的中位OS时间未达到。

## 讨论

Castleman病是一组罕见的、性质介于良性与恶性之间的淋巴组织增生性疾病，UCD的诊疗标准已较为成熟[3]，而MCD相对缺乏统一的诊疗规范[4]–[5]，故本研究中我们着重分析MCD患者的临床特征、治疗及预后。

本中心MCD患者中位发病年龄46岁，男女之比为2.3∶1。患者病理类型以浆细胞型居多（80％），而透明血管型（10％）和混合型（10％）占比较低。与此前国内报道类似[6]–[8]，患者以多发淋巴结肿大或发现肿物、伴有全身症状为主要临床表现。从实验室检查角度，患者多伴有血红蛋白和白蛋白水平的减低，多克隆免疫球蛋白、EPO、CRP等水平的升高，提示疾病消耗和炎症反应明显。此外，我们对合并免疫球蛋白升高的患者行相关鉴别诊断，均未发现克隆性增殖的证据，因此此类化验检查在初诊时虽可除外CD合并多发性骨髓瘤等浆细胞疾病，但对CD的诊断帮助不大。

CD的发病机制尚未完全明确。在一些iMCD患者中，IL-6升高可作为疾病发生的驱动因素。部分iMCD患者的症状严重程度与IL-6水平存在相关性：IL-6水平可在手术切除UCD患者肿大淋巴结或抗IL-6治疗后明显改善[9]–[11]，而在疾病进展时明显升高。赖玉梅等[11]的研究表明CD患者淋巴结组织IL-6表达水平与CD分型及系统症状密切相关。我们的研究显示，13例行细胞因子水平检测的MCD患者都至少有一种细胞因子水平的升高，一半以上的患者同时有≥3种细胞因子水平的升高。目前来看，CD的发生可能涉及多种细胞因子驱动[12]，进一步深入研究细胞及炎症因子在CD发病中的作用机制可能对药物研发及治疗选择提供理论基础。

司妥昔单抗（Siltuximab）是抗人IL-6受体的人源化单克隆抗体，能有效阻断IL-6信号通路。此前随机对照的研究表明[9]，司妥昔单抗用于治疗iMCD患者具有良好的安全性和有效性。我国MCD患者的HHV-8感染率仅占约9％[13]，故以iMCD为主[14]–[15]，因此抗IL-6治疗可作为MCD患者治疗方案的首选[16]。但目前司妥昔单抗及IL-6单抗妥珠单抗尚难以普遍应用，故传统化疗（联合或不联合利妥昔单抗）仍为MCD患者主要的治疗选择。虽然一部分患者在接受化疗后取得疾病缓解，但传统化疗复发率较高且不良反应明显，故不适合用于非重型的MCD患者。张路等[17]在2019年报道应用TCP（沙利度胺+环磷酰胺+泼尼松）口服方案治疗25例初诊iMCD患者，近半数患者达到了持续的肿瘤和症状学缓解，安全性及不良反应可控，为MCD患者治疗方案提供了新的选择。我们中心在2019年在5例患者中应用TCP方案，有1例患者用药1周后因皮疹改为TP方案，其他患者均未出现明显的不良反应。4例持续应用TCP方案的患者中，有1例保持疾病稳定状态，其余3例获得部分缓解的疗效。因本研究中MCD患者病例数较少，因此传统化疗组和免疫调节治疗组的优劣比较尚需继续收集病例扩大样本量后再次分析。

目前，MCD在我国的诊疗现状仍存在多种困难：MCD的临床表现异质性高，特异性低，多数患者就诊的过程辗转反复，不能得到及时的诊断治疗。确诊MCD极其依赖组织病理检查，这又对我国病理医师提出了更高的要求。对于临床及实验室检查高度符合CD而病理报告未明确诊断的患者，尽可能再次取淋巴结活检予以明确诊断，如无条件实行，应密切观察随访，并嘱患者必要时返院复查淋巴结病理，及时明确疾病转归，避免延误病情。
